# A new statistical distribution via the Phi-4 equation with its wide-ranging applications

**DOI:** 10.1371/journal.pone.0312458

**Published:** 2024-11-04

**Authors:** Yousef F. Alharbi, Ahmed M. T. Abd El-Bar, Mahmoud A. E. Abdelrahman, Ahmed M. Gemeay

**Affiliations:** 1 Department of Mathematics, College of Science, Taibah University, Madinah, Saudi Arabia; 2 Department of Mathematics, Faculty of Science, Tanta University, Tanta, Egypt; 3 Department of Mathematics, Faculty of Science, Mansoura University, Mansoura, Egypt; University of Hamburg: Universitat Hamburg, GERMANY

## Abstract

This paper presents a new framework based on nonlinear partial differential equations and statistics. For the nonlinear Phi-4 equation, the probability density function of the hyperbolic secant (HS) distribution has been obtained. Our model’s density has various shapes, including left-skewed, symmetric, and right-skewed. Eight distinct estimation approaches have been employed to estimate the parameters of our model. Additionally, the behavior of the HS model parameters was investigated using randomly generated data sets using these estimation techniques. Furthermore, we illustrate the applicability of the HS distribution for modeling real data by applying our results to real data. As a result, it is expected that our proposal will be of significant assistance to the community investigating new distributions based on hyperbolic functions and their applications to real-world data sets.

## 1 Introduction

Nonlinear partial differential equations (NPDEs) are tremendously important due to their numerous important applications [1–3]. In the current modern era of science, nonlinear phenomena is one of the most fascinating fields for researchers [4–6]. As a result, there is a lot of interest in extracting the solutions for NPDEs using various methodologies. Physical properties of finite or infinite dimensions, such as the conductivity field of a heterogeneous media, may be included in NPDEs-based models. These properties must have accurate values provided before the model can be utilized. In such cases, these parameters can be estimated using statistical techniques. These statistical methods operate on data and statistical hypotheses that describe the relationship between the data and the suggested mode of the NPDEs [7–9].

The Phi-4 equation is a well-known NPDE in the field of mathematical physics. This equation is investigated in various natural disciplines, including nuclear physics, superfluid, particle physics, plasma physics, and superfluid. The Phi-4 equation is given as follows [10]:

$$\begin{array}{c} \psi_{tt}-\psi_{xx}+a^{2}\psi +b\psi^{3}=0, \end{array}$$ (1)

where *ψ* = *ψ*(*x*, *t*) is a realistic wave function of *x*, *t*, whereas *a* and *b* are real-valued constants. The terms *ψ*_*tt*_, *ψ*_*xx*_ represent the effect of dissipation and the term *ψ*^3^ denotes the nonlinearity effect. The Phi-4 equation is a special form of the Klein–Gordon (KG) equation, related to the nonlinear Schrödinger equation [10]. Various quantum phenomena, such as matter waves dictating reality in the form of waves, which are the fundamental constituents of quantum mechanics, may be investigated using Phi-4 solutions. The Phi-4 equation may be used to examine ultrashort pulse propagation in optical transmission lines as well as particle physics events [11].

Numerous expansions of the lifespan distribution have been investigated in the statistical literature during the last two decades. Most extensions of classical distributions generated are algebraic, but even so, statistical distributions based on hyperbolic and trigonometric functions have recently received attention. These functions can be extremely useful in statistical studies due to their unlimited motivations and influence. The semi-hyperbolic distribution, a sub-type of the generalized hyperbolic distribution, was studied by [12]. Several generalized hyperbolic distribution types are covered by [13], along with how well they fit models to the data. A family of distributions known as the generalized secant hyperbolic distributions was established by Vaughan [14]. This family of symmetric distributions comprises numerous well-known distributions, including logistic and uniform distributions. Generally speaking, economics—more especially, the modeling of financial markets and risk management—uses the generalized hyperbolic distribution rather extensively. Among the distributions that made use of trigonometric and hyperbolic functions are beta trigonometric distribution [15], weighted cosine exponential distribution [16], the con-sine distribution [17], hyperbolic secant squared distribution [18], and a modified hyperbolic secant distribution [19].

Motivated by the previous debate, in addition to the ones currently available in the literature, a distribution based on a hyperbolic function has to be introduced; consequently, we give an extremely accurate link between the nonlinear partial differential equations and the generating distribution. We introduce the probability density function of the hyperbolic secant (HS) distribution corresponding to the nonlinear Phi-4 equation. As a practical example will show later, this model gives greater flexibility in modeling and assessing real data when compared to type IV generalized logistic, Gumbel, arctan Gumbel, logistic, normal, arctan normal, beta generalized logistic type IV, and type II beta generalized logistic distributions.

The rest of this work is given as follows. Section 2 presents the building block solution for the Phi-4 equation. In Sections 3, we create a new hyperbolic secant distribution to represent the solutions of the Phi-4 equation. In section 4, eight estimation methods are demonstrated to estimate the two parameters of the proposed distribution. Section 5 contains some numerical results. Section 6 explains how our key findings were applied to a real data set. Section 7 reports the conclusion.

## 2 Structure of building block solution

The goal of this part is to introduce the solution of the nonlinear Phi-4 equation. Utilizing the wave transformation

$$\begin{array}{c} \psi (x,t)=\Psi (\zeta ),\;\;\;\;\;\;\zeta =x-vt, \end{array}$$ (2)

v is the wave speed. Eq (1) becomes

$$\begin{array}{c} \left(v^{2}-1\right)\Psi^{\prime \prime }+b\Psi^{3}+a^{2}\Psi =0\;. \end{array}$$ (3)

In view of the unified solver [20], one of the vital solution of Eq (3) is given by:

$$\begin{array}{c} \Psi_{1,2}\left(\zeta \right)=\pm \sqrt{\frac{-2a^{2}}{b}}\;\text{sech}(\pm \sqrt{\frac{a^{2}}{\left(1-v^{2}\right)}}\zeta ). \end{array}$$ (4)

As a consequence, the solutions for Eq (1) are

$$\begin{array}{c} \psi_{1,2}\left(x,t\right)=\pm \sqrt{\frac{-2a^{2}}{b}}\;\text{sech}(\pm \sqrt{\frac{a^{2}}{\left(1-v^{2}\right)}}\left(x-vt\right)). \end{array}$$ (5)

## 3 Derivation of new kind of hyperbolic secant distribution

We will use a suitable statistical distribution as a model to solve the considered problem. Thus, a new hyperbolic secant (HS) distribution is derived as a solution to the Phi-4 Equation in (1). In general, the expression for the cdf of the new model is obtained by integrating the pdf of the same model. Now, we find the probability density function (pdf) using *ψ*_1,2_(*x*, *t* = 1), which is the present solution for the Phi-4 equation as expressed in Eq. (2.4). Therefore, we may have now

$$\begin{array}{c} \int_{-\infty }^{\infty }\sqrt{-\frac{2a^{2}}{b}}\text{Sech}\;[\sqrt{\frac{a^{2}}{1-\nu^{2}}}\left(x-\nu \right)]dx=\sqrt{2}\pi \sqrt{\frac{\nu^{2}-1}{b}}. \end{array}$$

Hence, considering $\frac{1}{\sqrt{2}\pi \sqrt{\frac{\nu^{2}-1}{b}}}$ as the normalizing constant, we obtain the pdf of our model as:

$$\begin{array}{c} f\left(x\right)=\frac{\left|a\right|}{\pi \sqrt{1-\nu^{2}}}\text{sech}\;(\sqrt{\frac{a^{2}}{1-\nu^{2}}}\left(x-\nu \right)),\;-\infty <x<\infty ,\;-1<\nu <1,-\infty <a<\infty ,\;a\neq 0, \end{array}$$ (6)

where *a* and *ν* are two parameters, and its cdf is

$$\begin{array}{c} F\left(x\right)=\frac{2({\text{tan}}^{-1}\;(e^{\frac{\nu |a|}{\sqrt{1-\nu^{2}}}})+{\text{tan}}^{-1}\;(\text{sech}(\frac{ax}{2\sqrt{1-\nu^{2}}})\;\text{sinh}\;(\frac{|a|\left(x-2\nu \right)}{2\sqrt{1-\nu^{2}}})))}{\pi }. \end{array}$$ (7)

**Fig 1:**
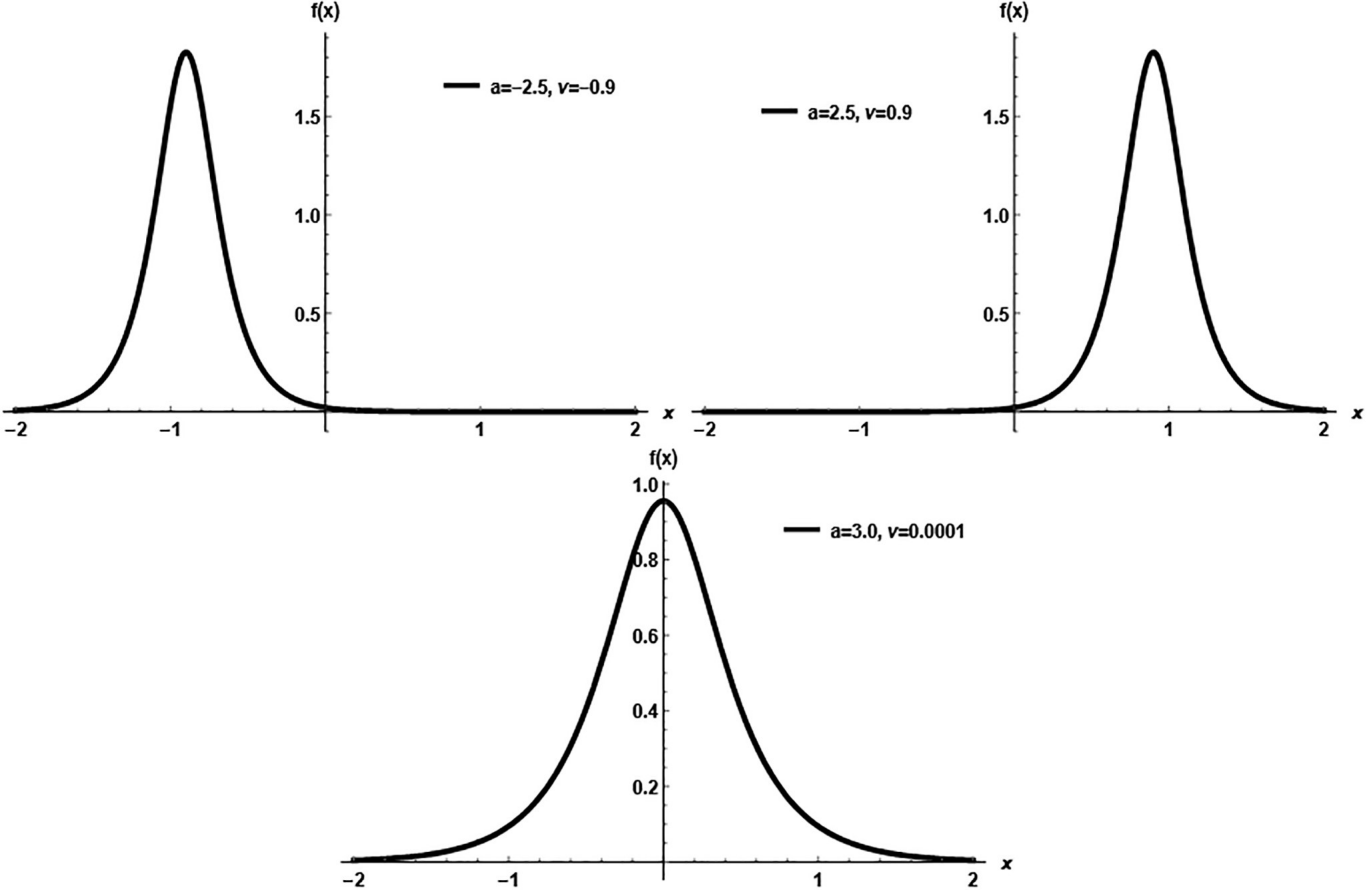
Possible PDF shapes of HS model.

The visual representations in Fig 1 elucidate the versatility of the pdf within the HS model across a range of parameter values. These plots illustrate that the proposed pdf is not confined to a singular form but exhibits a rich diversity in shape, including symmetric, left-skewed, and right-skewed distributions. This variability is indicative of the model’s adaptability to different statistical scenarios. It underscores its potential applicability in various contexts where data distributions may deviate from standard symmetrical patterns.

**Remark 1** *The HS distribution’s mean is defined as follows*:

E[x]=21-ν2|a|e-ν|a|1-ν2πa2×(e2ν|a|1-ν2F32(12,12,1;32,32;-e2ν|a|1-ν2)-F32(12,12,1;32,32;-e-2ν|a|1-ν2)), (8)

*where the generalized hypergeometric function is defined as*

Fpq(a1,…,ap;b1,…,bq;z)=∑n=0∞(a1)n…(ap)n(b1)n…(bq)nznn!, (9)

*where* (*a*)_*n*_ *is the Pochammer symbol*, (*a*)_*n*_ = *a*(*a* + 1)(*a* + 2) ⋯ (*a* + *n* − 1), *n* ≥ 1.

*Further, the second-order moment of the HS model is found as*

$$\begin{array}{c} E\left[x^{2}\right]=\frac{\left(1-\nu^{2})e^{-\frac{a\nu }{\sqrt{1-\nu^{2}}}}(e^{\frac{2a\nu }{\sqrt{1-\nu^{2}}}}\Phi (-e^{\frac{2a\nu }{\sqrt{1-\nu^{2}}}},3,\frac{1}{2})+\Phi (-e^{-\frac{2a\nu }{\sqrt{1-\nu^{2}}}},3,\frac{1}{2})\right)}{2\pi a^{2}}, \end{array}$$ (10)

*where the Lerch transcendent function* Φ(*z*, *s*, *a*) *is defined as*

$$\begin{array}{c} \Phi \left(z,s,a\right)=\sum_{k=0}^{\infty }\frac{z^{k}}{{\left(a+k\right)}^{s}},\;\left|z\right|<1. \end{array}$$

From the above moments, we can obtain the variance of the HS model.

## 4 Estimation methods

In this section, we aim to find estimators of parameters for our proposed model $\hat{\nu }$ and $\hat{a}$ by different estimation methods. In a parametric estimation problem, estimation methods are generic procedures that can be utilized to derive estimators. These methods generally calculate the parameters of a distribution or model by maximizing or minimizing an objective function. For more details about estimation methods, the reader is referred to [21, 22]. In the current work, we will consider eight different estimation methods to estimate the parameters of our model as follows.

The first method is the maximum likelihood estimation method (EM1). Using this method, our proposed model estimators $\hat{\nu }$ and $\hat{a}$ are obtained by maximizing the following equation

$$\begin{array}{c} l=\sum_{i=1}^{n}\;\text{log}\;(\text{sech}(\sqrt{\frac{a^{2}}{1-\nu^{2}}}(x_{i}-\nu )))+n\;\text{log}\;(\frac{\left|a\right|}{\pi \sqrt{1-\nu^{2}}}). \end{array}$$

By Anderson-Darling estimation method (EM2), our proposed model estimators $\hat{\nu }$ and $\hat{a}$ are computed by minimizing the following equation

$$\begin{array}{cc} A & =-n-\frac{1}{n}\sum_{i=1}^{n}\left(2i-1\right)\left[\text{log}\;F\left(x_{i}\right)+\text{log}\;S\left(x_{i}\right)\right], \end{array}$$

where *S*(*x*_*i*_) refers to the survival function of our proposed model.

The third method is Cramér-von Mises estimation method (EM3). In this method, our proposed model estimators $\hat{\nu }$ and $\hat{a}$ are found by minimizing the following equation

$$\begin{array}{c} C=-\frac{1}{12n}+\sum_{i=1}^{n}{\left[F\left(x_{i}\right)-\frac{2i-1}{2n}\right]}^{2}. \end{array}$$

In the fourth method, the maximum product of the spacings estimation method (EM4) is used to estimate $\hat{\nu }$ and $\hat{a}$. It is calculated by maximizing the following equation

$$\begin{array}{c} M=\frac{1}{n+1}\sum_{i=1}^{n+1}\;\text{log}\;T_{i}, \end{array}$$

where

$$\begin{array}{c} T_{i}=F(x_{\left(i\right)})-F(x_{\left(i-1\right)}). \end{array}$$

The fifth method is the least-squares estimation method (EM5). Utilizing this method, the estimators of $\hat{\nu }$ and $\hat{a}$ are worked out by minimizing the following equation

$$\begin{array}{cc} V & =\sum_{i=1}^{n}{\left[F\left(x_{i}\right)-\frac{i}{n+1}\right]}^{2}. \end{array}$$

In the sixth method, the percentile estimation method (EM6) is employed to find $\hat{\nu }$ and $\hat{a}$ by minimizing the following equation

$$\begin{array}{c} PC=\sum_{i=1}^{n}{\left[x_{i}-Q\left(e_{i}\right)\right]}^{2}, \end{array}$$

where *Q*(*e*_*i*_) refers to the quantile function of our proposed model.

Our proposed model estimators $\hat{\nu }$ and $\hat{a}$ are obtained using the right-tail Anderson-Darling estimation method (EM7). It is computed by minimizing the following equation

$$\begin{array}{c} R=\frac{n}{2}-2\sum_{i=1}^{n}F(x_{i})-\frac{1}{n}\sum_{i=1}^{n}(2i-1)\;\text{log}\;S(x_{n+1-i}). \end{array}$$

In the last method, the weighted least-squares estimation method (EM8) is considered to calculate the estimators of $\hat{\nu }$ and $\hat{a}$ by minimizing the following equation

$$\begin{array}{c} W=\sum_{i=1}^{n}\frac{{\left(n+1\right)}^{2}\left(n+2\right)}{i(n-i+1)}{\left[F\left(x_{i}\right)-\frac{i}{n+1}\right]}^{2}. \end{array}$$

The performance of these estimation methods will be discussed in the coming section using some numerical examples. The performance of estimation methods may be varied from one model to another. In other words, it might be the performance of an estimation method in a problem is the best for finding the parameters of the models, but in another problem, it is not.

## 5 Numerical simulation

This section studies the behavior of all estimation methods determined in Section 4. Using our proposed model, we randomly generated data sets and found proposed model estimators using these estimation methods. For comparison purposes, we use the following three measures to assess the performance of the estimation methods as follows:

1. The average of absolute bias (BIAS), which is computed by $|Bias(\hat{\Xi })|=\frac{1}{M}\sum_{i=1}^{M}\left|\hat{\Xi }-\Xi \right|$.
2. The mean squared errors (MSE), which is found using $MSE=\frac{1}{M}\sum_{i=1}^{M}{\left(\hat{\Xi }-\Xi \right)}^{2}$.
3. The mean absolute relative errors (MRE), which is calculated by $MRE=\frac{1}{M}\sum_{i=1}^{M}\left|\hat{\Xi }-\Xi \right|/\Xi ,\Xi =(\nu ,a)$.

The other aim of this simulation study is to know the best estimation method to calculate our proposed model estimators. We perform this simulation by generating random samples from our model with different sizes and determining the measures we use. Then, we repeat these steps thousands of times.

**Fig 2:**
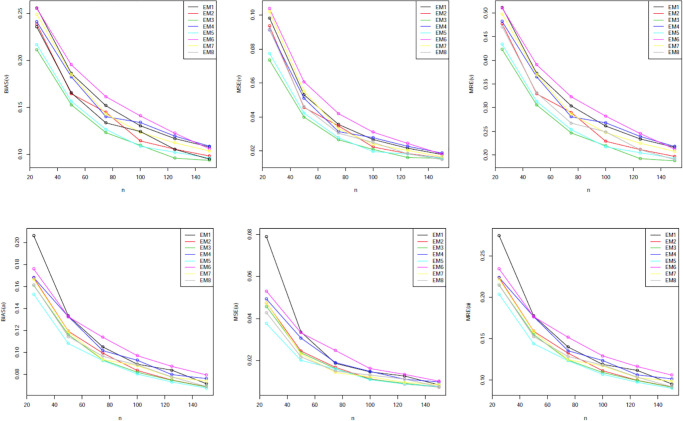
Graphical representation of BIAS, MSE; MRE values of Table 1.

Our simulation values are presented in Tables 1–5. The graphical representations provided in Figs 2 through 6 offer a visual depiction of the data elucidated within Tables 1 to 5. A discernible trend emerges across these figures, demonstrating a consistent decline in all measured variables with the increment in sample size. The power of any value relates to its rank when comparing all estimating methodologies. Our estimator’s partial and overall rankings are displayed in Table 6.

**Fig 3:**
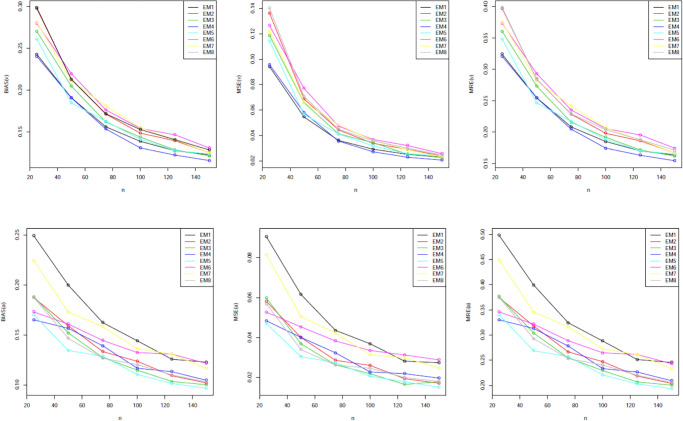
Graphical representation of BIAS, MSE; MRE values of Table 2.

**Fig 4:**
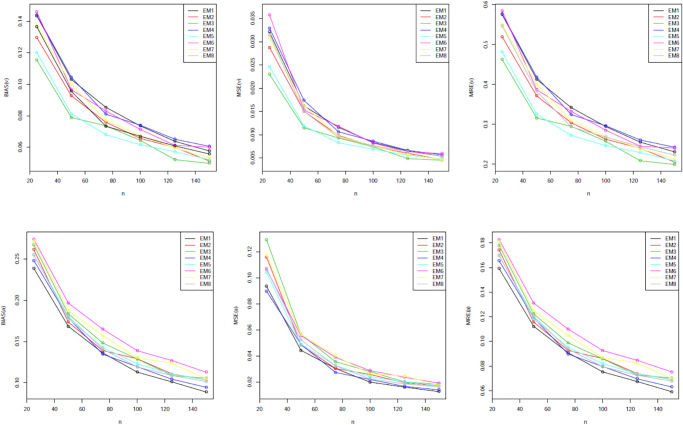
Graphical representation of BIAS, MSE; MRE values of Table 3.

**Fig 5:**
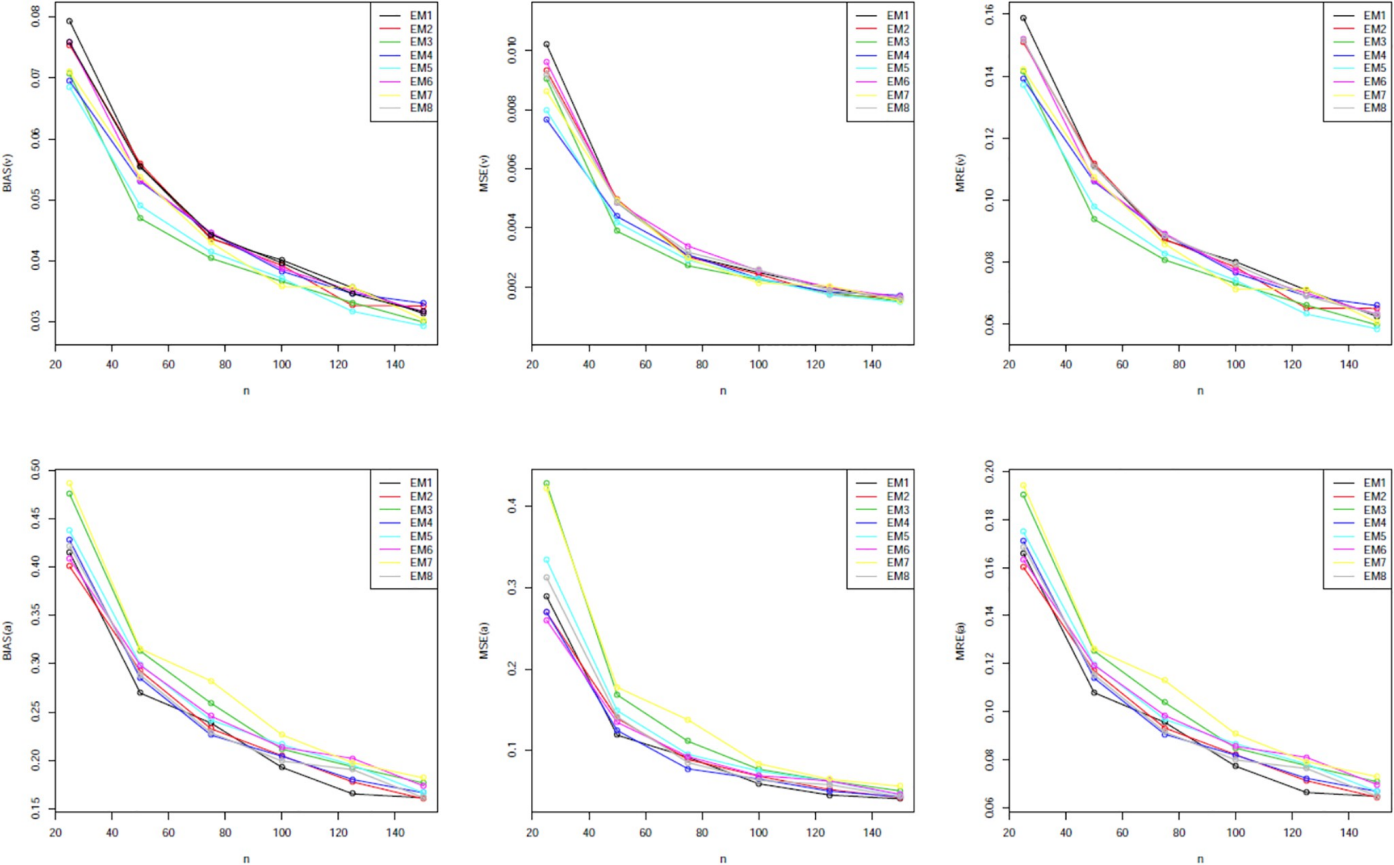
Graphical representation of BIAS, MSE; MRE values of Table 4.

**Fig 6:**
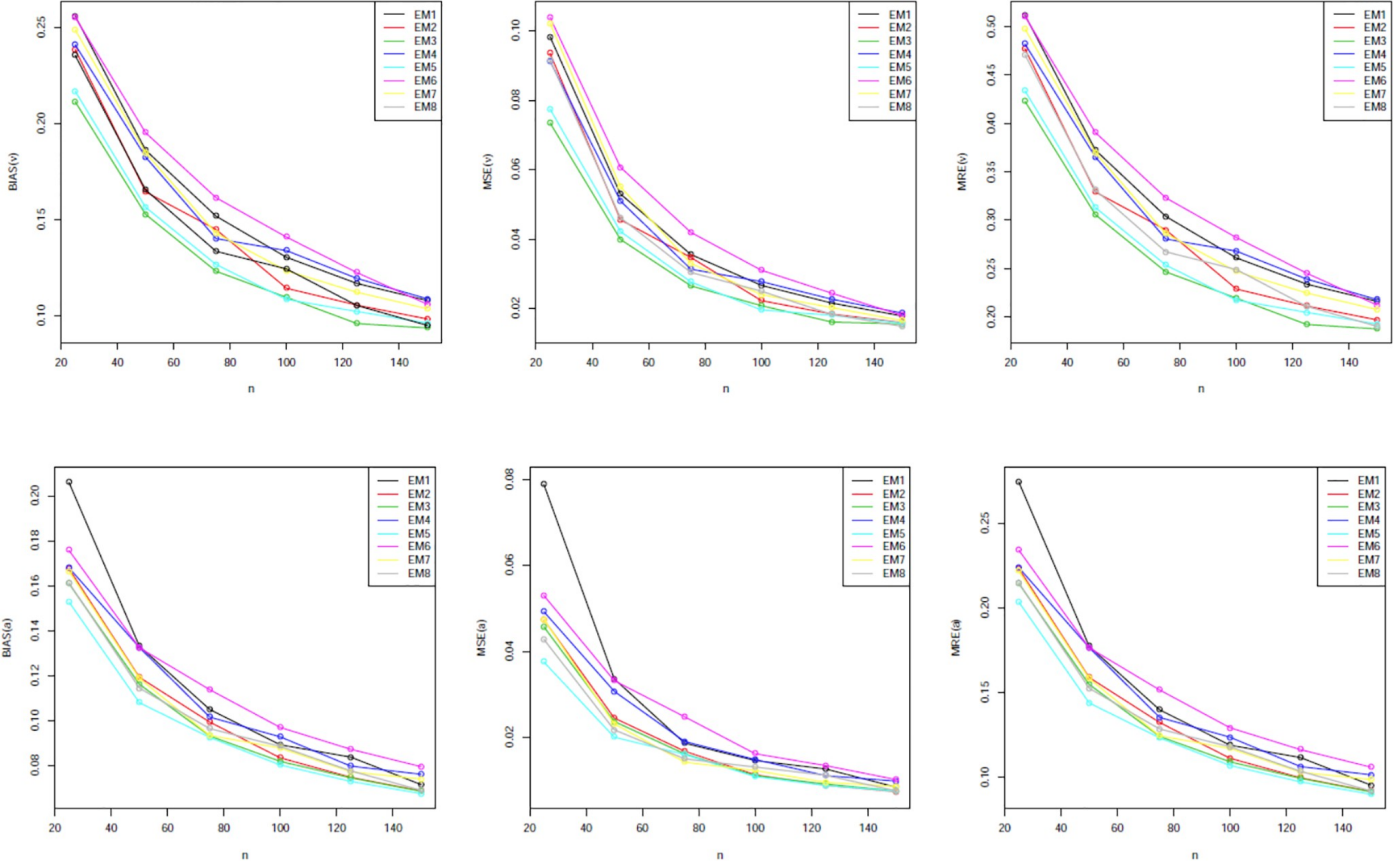
Graphical representation of BIAS, MSE; MRE values of Table 5.

**Table 1:** Simulation values of BIAS, MSE; MRE for (*ν* = −0.5, *a* = −0.75).

n	Est.	Est. Par.	*EM*1	*EM*2	*EM*3	*EM*4	*EM*5	*EM*6	*EM*7	*EM*8
25	BIAS	$\hat{\nu }$	0.256^{8}^	0.23858^{4}^	0.2114^{1}^	0.24101^{5}^	0.21684^{2}^	0.25532^{7}^	0.24883^{6}^	0.23548^{3}^
		$\hat{a}$	0.20607^{8}^	0.16765^{5}^	0.16104^{2}^	0.16807^{6}^	0.1529^{1}^	0.17585^{7}^	0.16652^{4}^	0.16135^{3}^
	MSE	$\hat{\nu }$	0.09826^{6}^	0.09348^{5}^	0.07349^{1}^	0.09128^{3}^	0.07748^{2}^	0.10384^{8}^	0.10216^{7}^	0.09154^{4}^
		$\hat{a}$	0.07893^{8}^	0.04741^{4}^	0.04576^{3}^	0.04929^{6}^	0.03757^{1}^	0.05305^{7}^	0.0475^{5}^	0.04284^{2}^
	MRE	$\hat{\nu }$	0.512^{8}^	0.47716^{4}^	0.42281^{1}^	0.48202^{5}^	0.43369^{2}^	0.51065^{7}^	0.49767^{6}^	0.47096^{3}^
		$\hat{a}$	0.27476^{8}^	0.22354^{5}^	0.21472^{2}^	0.22409^{6}^	0.20386^{1}^	0.23447^{7}^	0.22202^{4}^	0.21513^{3}^
	∑ *Ranks*		46^{8}^	27^{4}^	10^{2}^	31^{5}^	9^{1}^	43^{7}^	32^{6}^	18^{3}^
50	BIAS	$\hat{\nu }$	0.18615^{7}^	0.16444^{3}^	0.15274^{1}^	0.18253^{5}^	0.15643^{2}^	0.19521^{8}^	0.18479^{6}^	0.16549^{4}^
		$\hat{a}$	0.1332^{8}^	0.11936^{5}^	0.11627^{3}^	0.1326^{7}^	0.1081^{1}^	0.13253^{6}^	0.11886^{4}^	0.11456^{2}^
	MSE	$\hat{\nu }$	0.05304^{6}^	0.04562^{3}^	0.03997^{1}^	0.05103^{5}^	0.04226^{2}^	0.06072^{8}^	0.05511^{7}^	0.04619^{4}^
		$\hat{a}$	0.03363^{8}^	0.02451^{5}^	0.0237^{4}^	0.03073^{6}^	0.02006^{1}^	0.03321^{7}^	0.02314^{3}^	0.02175^{2}^
	MRE	$\hat{\nu }$	0.3723^{7}^	0.32888^{3}^	0.30548^{1}^	0.36507^{5}^	0.31285^{2}^	0.39043^{8}^	0.36957^{6}^	0.33097^{4}^
		$\hat{a}$	0.1776^{8}^	0.15915^{5}^	0.15503^{3}^	0.1768^{7}^	0.14413^{1}^	0.17671^{6}^	0.15848^{4}^	0.15275^{2}^
	∑ *Ranks*		44^{8}^	24^{4}^	13^{2}^	35^{6}^	9^{1}^	43^{7}^	30^{5}^	18^{3}^
75	BIAS	$\hat{\nu }$	0.1518^{7}^	0.14485^{6}^	0.12314^{1}^	0.14014^{4}^	0.12665^{2}^	0.16141^{8}^	0.1429^{5}^	0.13352^{3}^
		$\hat{a}$	0.10503^{7}^	0.09942^{5}^	0.09324^{2}^	0.10165^{6}^	0.09261^{1}^	0.11384^{8}^	0.09353^{3}^	0.09652^{4}^
	MSE	$\hat{\nu }$	0.03563^{7}^	0.03477^{6}^	0.02661^{1}^	0.03139^{4}^	0.02774^{2}^	0.04199^{8}^	0.03306^{5}^	0.03046^{3}^
		$\hat{a}$	0.01864^{6}^	0.01678^{5}^	0.01614^{4}^	0.01904^{7}^	0.01601^{3}^	0.02479^{8}^	0.01424^{1}^	0.015^{2}^
	MRE	$\hat{\nu }$	0.30359^{7}^	0.28969^{6}^	0.24628^{1}^	0.28027^{4}^	0.2533^{2}^	0.32282^{8}^	0.28581^{5}^	0.26703^{3}^
		$\hat{a}$	0.14004^{7}^	0.13256^{5}^	0.12432^{2}^	0.13554^{6}^	0.12347^{1}^	0.15178^{8}^	0.12471^{3}^	0.12869^{4}^
	∑ *Ranks*		41^{7}^	33^{6}^	11^{1.5}^	31^{5}^	11^{1.5}^	48^{8}^	22^{4}^	19^{3}^
100	BIAS	$\hat{\nu }$	0.1304^{6}^	0.1143^{3}^	0.1096^{2}^	0.13388^{7}^	0.10852^{1}^	0.14091^{8}^	0.12339^{4}^	0.12435^{5}^
		$\hat{a}$	0.08935^{6}^	0.08356^{3}^	0.08198^{2}^	0.09289^{7}^	0.08036^{1}^	0.09707^{8}^	0.08791^{4}^	0.08866^{5}^
	MSE	$\hat{\nu }$	0.02675^{6}^	0.02237^{3}^	0.02092^{2}^	0.02784^{7}^	0.01969^{1}^	0.0311^{8}^	0.0241^{4}^	0.0251^{5}^
		$\hat{a}$	0.01462^{6}^	0.01131^{3}^	0.01111^{2}^	0.01485^{7}^	0.01087^{1}^	0.0162^{8}^	0.01228^{4}^	0.01311^{5}^
	MRE	$\hat{\nu }$	0.26079^{6}^	0.2286^{3}^	0.2192^{2}^	0.26777^{7}^	0.21705^{1}^	0.28182^{8}^	0.24678^{4}^	0.2487^{5}^
		$\hat{a}$	0.11914^{6}^	0.11141^{3}^	0.1093^{2}^	0.12385^{7}^	0.10715^{1}^	0.12943^{8}^	0.11721^{4}^	0.11821^{5}^
	∑ *Ranks*		36^{6}^	18^{3}^	12^{2}^	42^{7}^	6^{1}^	48^{8}^	24^{4}^	30^{5}^
125	BIAS	$\hat{\nu }$	0.11679^{6}^	0.10554^{4}^	0.09595^{1}^	0.11942^{7}^	0.10222^{2}^	0.12244^{8}^	0.11223^{5}^	0.10534^{3}^
		$\hat{a}$	0.08388^{7}^	0.07492^{3}^	0.07466^{2}^	0.07989^{6}^	0.07309^{1}^	0.08737^{8}^	0.07737^{4}^	0.07777^{5}^
	MSE	$\hat{\nu }$	0.02166^{6}^	0.0185^{4}^	0.01621^{1}^	0.02279^{7}^	0.01814^{2}^	0.0245^{8}^	0.02037^{5}^	0.0184^{3}^
		$\hat{a}$	0.01265^{7}^	0.00881^{2}^	0.00914^{3}^	0.01109^{5}^	0.0088^{1}^	0.01344^{8}^	0.00956^{4}^	0.01117^{6}^
	MRE	$\hat{\nu }$	0.23358^{6}^	0.21108^{4}^	0.1919^{1}^	0.23885^{7}^	0.20444^{2}^	0.24488^{8}^	0.22446^{5}^	0.21068^{3}^
		$\hat{a}$	0.11184^{7}^	0.09989^{3}^	0.09955^{2}^	0.10651^{6}^	0.09745^{1}^	0.1165^{8}^	0.10316^{4}^	0.1037^{5}^
	∑ *Ranks*		39^{7}^	20^{3}^	10^{2}^	38^{6}^	9^{1}^	48^{8}^	27^{5}^	25^{4}^
150	BIAS	$\hat{\nu }$	0.10793^{7}^	0.09833^{4}^	0.09365^{1}^	0.1088^{8}^	0.0962^{3}^	0.10598^{6}^	0.10367^{5}^	0.09498^{2}^
		$\hat{a}$	0.07163^{5}^	0.0689^{3}^	0.06867^{2}^	0.07626^{7}^	0.06761^{1}^	0.07963^{8}^	0.07416^{6}^	0.06913^{4}^
	MSE	$\hat{\nu }$	0.01795^{6}^	0.01603^{4}^	0.01566^{2}^	0.01877^{8}^	0.01586^{3}^	0.01811^{7}^	0.0166^{5}^	0.01494^{1}^
		$\hat{a}$	0.0084^{5}^	0.00736^{1}^	0.00755^{3.5}^	0.00984^{7}^	0.00749^{2}^	0.01019^{8}^	0.0086^{6}^	0.00755^{3.5}^
	MRE	$\hat{\nu }$	0.21587^{7}^	0.19665^{4}^	0.18731^{1}^	0.2176^{8}^	0.1924^{3}^	0.21196^{6}^	0.20735^{5}^	0.18996^{2}^
		$\hat{a}$	0.09551^{5}^	0.09186^{3}^	0.09156^{2}^	0.10168^{7}^	0.09015^{1}^	0.10617^{8}^	0.09887^{6}^	0.09217^{4}^
	∑ *Ranks*		35^{6}^	19^{4}^	11.5^{1}^	45^{8}^	13^{2}^	43^{7}^	33^{5}^	16.5^{3}^

**Table 2:** Simulation values of BIAS, MSE; MRE for (*ν* = 0.75, *a* = 0.5).

n	Est.	Est. Par.	*EM*1	*EM*2	*EM*3	*EM*4	*EM*5	*EM*6	*EM*7	*EM*8
25	BIAS	$\hat{\nu }$	0.24295^{2}^	0.29753^{7}^	0.27027^{4}^	0.24039^{1}^	0.26117^{3}^	0.27995^{5}^	0.28131^{6}^	0.29868^{8}^
		$\hat{a}$	0.2492^{8}^	0.18734^{4}^	0.1883^{6}^	0.16513^{1}^	0.17015^{2}^	0.17304^{3}^	0.22451^{7}^	0.18756^{5}^
	MSE	$\hat{\nu }$	0.0942^{1}^	0.13638^{7}^	0.11868^{4}^	0.09585^{2}^	0.1143^{3}^	0.12652^{6}^	0.1217^{5}^	0.1402^{8}^
		$\hat{a}$	0.0905^{8}^	0.05808^{5}^	0.05978^{6}^	0.04838^{2}^	0.04711^{1}^	0.0527^{3}^	0.08155^{7}^	0.05685^{4}^
	MRE	$\hat{\nu }$	0.32393^{2}^	0.3967^{7}^	0.36036^{4}^	0.32052^{1}^	0.34823^{3}^	0.37327^{5}^	0.37509^{6}^	0.39824^{8}^
		$\hat{a}$	0.49841^{8}^	0.37469^{4}^	0.3766^{6}^	0.33026^{1}^	0.34029^{2}^	0.34609^{3}^	0.44902^{7}^	0.37512^{5}^
	∑ *Ranks*		29^{4}^	34^{6}^	30^{5}^	8^{1}^	14^{2}^	25^{3}^	38^{7.5}^	38^{7.5}^
50	BIAS	$\hat{\nu }$	0.19093^{3}^	0.21306^{7}^	0.20482^{4}^	0.19075^{2}^	0.18472^{1}^	0.21972^{8}^	0.21098^{5}^	0.2128^{6}^
		$\hat{a}$	0.1998^{8}^	0.15913^{5}^	0.15205^{3}^	0.1566^{4}^	0.13449^{1}^	0.16089^{6}^	0.17297^{7}^	0.14648^{2}^
	MSE	$\hat{\nu }$	0.05456^{1}^	0.0688^{6}^	0.06584^{4}^	0.05807^{3}^	0.05658^{2}^	0.07713^{8}^	0.06618^{5}^	0.07077^{7}^
		$\hat{a}$	0.0616^{8}^	0.04004^{4}^	0.03691^{3}^	0.04008^{5}^	0.03039^{1}^	0.04538^{6}^	0.0505^{7}^	0.03396^{2}^
	MRE	$\hat{\nu }$	0.25457^{3}^	0.28408^{7}^	0.27309^{4}^	0.25434^{2}^	0.2463^{1}^	0.29295^{8}^	0.28131^{5}^	0.28373^{6}^
		$\hat{a}$	0.3996^{8}^	0.31827^{5}^	0.3041^{3}^	0.31321^{4}^	0.26898^{1}^	0.32179^{6}^	0.34593^{7}^	0.29297^{2}^
	∑ *Ranks*		31^{5}^	34^{6}^	21^{3}^	20^{2}^	7^{1}^	42^{8}^	36^{7}^	25^{4}^
75	BIAS	$\hat{\nu }$	0.15606^{2}^	0.17124^{5}^	0.16176^{3}^	0.1534^{1}^	0.16274^{4}^	0.17627^{7}^	0.18104^{8}^	0.17168^{6}^
		$\hat{a}$	0.16232^{8}^	0.13334^{4}^	0.12736^{2}^	0.13916^{5}^	0.12871^{3}^	0.14448^{6}^	0.15827^{7}^	0.12667^{1}^
	MSE	$\hat{\nu }$	0.03608^{2}^	0.04439^{5}^	0.04126^{4}^	0.03557^{1}^	0.04115^{3}^	0.04734^{7}^	0.0483^{8}^	0.04483^{6}^
		$\hat{a}$	0.04362^{8}^	0.02861^{4}^	0.02634^{2}^	0.03238^{5}^	0.02748^{3}^	0.03831^{6}^	0.04228^{7}^	0.02629^{1}^
	MRE	$\hat{\nu }$	0.20808^{2}^	0.22832^{5}^	0.21568^{3}^	0.20453^{1}^	0.21699^{4}^	0.23502^{7}^	0.24139^{8}^	0.2289^{6}^
		$\hat{a}$	0.32464^{8}^	0.26668^{4}^	0.25472^{2}^	0.27832^{5}^	0.25741^{3}^	0.28895^{6}^	0.31653^{7}^	0.25334^{1}^
	∑ *Ranks*		30^{6}^	27^{5}^	16^{1}^	18^{2}^	20^{3}^	39^{7}^	45^{8}^	21^{4}^
100	BIAS	$\hat{\nu }$	0.13861^{2}^	0.14845^{5}^	0.14365^{4}^	0.13076^{1}^	0.14115^{3}^	0.15413^{7}^	0.15484^{8}^	0.15275^{6}^
		$\hat{a}$	0.14412^{8}^	0.12363^{5}^	0.11478^{2}^	0.11669^{3}^	0.11046^{1}^	0.13241^{6}^	0.13633^{7}^	0.12031^{4}^
	MSE	$\hat{\nu }$	0.02922^{2}^	0.03377^{4}^	0.03437^{5}^	0.02715^{1}^	0.03177^{3}^	0.03684^{8}^	0.03605^{7}^	0.03574^{6}^
		$\hat{a}$	0.03693^{8}^	0.02607^{5}^	0.02193^{2}^	0.02278^{3}^	0.02064^{1}^	0.03354^{7}^	0.03146^{6}^	0.02463^{4}^
	MRE	$\hat{\nu }$	0.18481^{2}^	0.19793^{5}^	0.19153^{4}^	0.17435^{1}^	0.1882^{3}^	0.2055^{7}^	0.20645^{8}^	0.20367^{6}^
		$\hat{a}$	0.28823^{8}^	0.24725^{5}^	0.22957^{2}^	0.23339^{3}^	0.22092^{1}^	0.26483^{6}^	0.27266^{7}^	0.24062^{4}^
	∑ *Ranks*		30^{5.5}^	29^{4}^	19^{3}^	12^{1.5}^	12^{1.5}^	41^{7}^	43^{8}^	30^{5.5}^
125	BIAS	$\hat{\nu }$	0.12745^{2}^	0.13944^{5}^	0.1286^{4}^	0.12224^{1}^	0.12756^{3}^	0.14651^{8}^	0.14056^{6}^	0.14068^{7}^
		$\hat{a}$	0.12571^{6}^	0.1092^{3}^	0.10342^{2}^	0.11327^{5}^	0.1015^{1}^	0.13079^{8}^	0.13076^{7}^	0.11007^{4}^
	MSE	$\hat{\nu }$	0.02511^{2}^	0.0291^{6}^	0.0253^{3}^	0.02283^{1}^	0.02561^{4}^	0.03207^{8}^	0.02882^{5}^	0.02996^{7}^
		$\hat{a}$	0.02821^{6}^	0.01942^{3}^	0.01655^{1}^	0.02192^{5}^	0.01788^{2}^	0.03129^{8}^	0.02994^{7}^	0.02004^{4}^
	MRE	$\hat{\nu }$	0.16994^{2}^	0.18593^{5}^	0.17146^{4}^	0.16299^{1}^	0.17009^{3}^	0.19534^{8}^	0.18741^{6}^	0.18757^{7}^
		$\hat{a}$	0.25143^{6}^	0.21841^{3}^	0.20685^{2}^	0.22654^{5}^	0.203^{1}^	0.26157^{8}^	0.26152^{7}^	0.22013^{4}^
	∑ *Ranks*		24^{4}^	25^{5}^	16^{2}^	18^{3}^	14^{1}^	48^{8}^	38^{7}^	33^{6}^
150	BIAS	$\hat{\nu }$	0.12259^{3}^	0.12503^{6}^	0.12123^{2}^	0.11568^{1}^	0.12368^{4}^	0.13069^{8}^	0.12487^{5}^	0.12832^{7}^
		$\hat{a}$	0.12281^{8}^	0.10197^{3}^	0.10023^{2}^	0.10464^{5}^	0.09666^{1}^	0.12152^{7}^	0.11662^{6}^	0.1031^{4}^
	MSE	$\hat{\nu }$	0.02272^{2}^	0.02395^{5}^	0.02305^{4}^	0.0206^{1}^	0.02416^{6}^	0.02567^{8}^	0.02296^{3}^	0.02477^{7}^
		$\hat{a}$	0.0274^{7}^	0.01712^{2}^	0.01789^{3}^	0.0197^{5}^	0.0151^{1}^	0.02883^{8}^	0.02463^{6}^	0.01799^{4}^
	MRE	$\hat{\nu }$	0.16345^{3}^	0.1667^{6}^	0.16164^{2}^	0.15424^{1}^	0.1649^{4}^	0.17426^{8}^	0.16649^{5}^	0.17109^{7}^
		$\hat{a}$	0.24561^{8}^	0.20394^{3}^	0.20047^{2}^	0.20928^{5}^	0.19331^{1}^	0.24304^{7}^	0.23324^{6}^	0.2062^{4}^
	∑ *Ranks*		31^{5.5}^	25^{4}^	15^{1}^	18^{3}^	17^{2}^	46^{8}^	31^{5.5}^	33^{7}^

**Table 3:** Simulation values of BIAS, MSE; MRE for (*ν* = −0.25, *a* = 1.5).

n	Est.	Est. Par.	*EM*1	*EM*2	*EM*3	*EM*4	*EM*5	*EM*6	*EM*7	*EM*8
25	BIAS	$\hat{\nu }$	0.14358^{6}^	0.12972^{3}^	0.11544^{1}^	0.14398^{7}^	0.1202^{2}^	0.14607^{8}^	0.13723^{5}^	0.13653^{4}^
		$\hat{a}$	0.23878^{1}^	0.26159^{5}^	0.26731^{6}^	0.24824^{2}^	0.25525^{4}^	0.27406^{8}^	0.27128^{7}^	0.25463^{3}^
	MSE	$\hat{\nu }$	0.03209^{6}^	0.02879^{3}^	0.02299^{1}^	0.03293^{7}^	0.02464^{2}^	0.03578^{8}^	0.03078^{4}^	0.03151^{5}^
		$\hat{a}$	0.09369^{2}^	0.11617^{6}^	0.12943^{8}^	0.08984^{1}^	0.10418^{3}^	0.10669^{4}^	0.11768^{7}^	0.10726^{5}^
	MRE	$\hat{\nu }$	0.57432^{6}^	0.51888^{3}^	0.46176^{1}^	0.57593^{7}^	0.48081^{2}^	0.58429^{8}^	0.5489^{5}^	0.54611^{4}^
		$\hat{a}$	0.15919^{1}^	0.17439^{5}^	0.17821^{6}^	0.1655^{2}^	0.17017^{4}^	0.18271^{8}^	0.18085^{7}^	0.16975^{3}^
	∑ *Ranks*		22^{2}^	25^{5}^	23^{3}^	26^{6}^	17^{1}^	44^{8}^	35^{7}^	24^{4}^
50	BIAS	$\hat{\nu }$	0.10309^{7}^	0.0927^{3}^	0.07889^{1}^	0.1045^{8}^	0.08124^{2}^	0.09659^{5}^	0.09988^{6}^	0.09559^{4}^
		$\hat{a}$	0.16845^{1}^	0.17366^{2}^	0.18359^{6}^	0.17887^{4}^	0.1779^{3}^	0.19673^{8}^	0.18757^{7}^	0.18041^{5}^
	MSE	$\hat{\nu }$	0.01621^{6}^	0.01505^{4}^	0.01151^{1}^	0.01739^{8}^	0.01189^{2}^	0.0153^{5}^	0.0163^{7}^	0.01503^{3}^
		$\hat{a}$	0.04434^{1}^	0.04841^{2}^	0.05659^{8}^	0.04899^{4}^	0.04896^{3}^	0.05566^{6}^	0.05649^{7}^	0.05235^{5}^
	MRE	$\hat{\nu }$	0.41235^{7}^	0.37079^{3}^	0.31557^{1}^	0.418^{8}^	0.32497^{2}^	0.38634^{5}^	0.39951^{6}^	0.38238^{4}^
		$\hat{a}$	0.1123^{1}^	0.11578^{2}^	0.12239^{6}^	0.11924^{4}^	0.1186^{3}^	0.13116^{8}^	0.12504^{7}^	0.12027^{5}^
	∑ *Ranks*		23^{3.5}^	16^{2}^	23^{3.5}^	36^{6}^	15^{1}^	37^{7}^	40^{8}^	26^{5}^
75	BIAS	$\hat{\nu }$	0.08538^{8}^	0.07589^{4}^	0.07362^{3}^	0.08101^{6}^	0.06802^{1}^	0.08304^{7}^	0.07717^{5}^	0.07334^{2}^
		$\hat{a}$	0.13622^{2}^	0.139^{3}^	0.14821^{6}^	0.13464^{1}^	0.14278^{5}^	0.16504^{8}^	0.15735^{7}^	0.1407^{4}^
	MSE	$\hat{\nu }$	0.01161^{7}^	0.00986^{5}^	0.00931^{2}^	0.01065^{6}^	0.00829^{1}^	0.01185^{8}^	0.0097^{4}^	0.00944^{3}^
		$\hat{a}$	0.03039^{3}^	0.03038^{2}^	0.03562^{6}^	0.02734^{1}^	0.03269^{5}^	0.03897^{7}^	0.04021^{8}^	0.03237^{4}^
	MRE	$\hat{\nu }$	0.34151^{8}^	0.30357^{4}^	0.29448^{3}^	0.32402^{6}^	0.27207^{1}^	0.33217^{7}^	0.30866^{5}^	0.29337^{2}^
		$\hat{a}$	0.09081^{2}^	0.09267^{3}^	0.09881^{6}^	0.08976^{1}^	0.09519^{5}^	0.11003^{8}^	0.1049^{7}^	0.0938^{4}^
	∑ *Ranks*		30^{6}^	21^{3.5}^	26^{5}^	21^{3.5}^	18^{1}^	45^{8}^	36^{7}^	19^{2}^
100	BIAS	$\hat{\nu }$	0.07352^{7}^	0.06561^{3}^	0.06433^{2}^	0.07401^{8}^	0.06159^{1}^	0.07126^{6}^	0.06718^{5}^	0.06694^{4}^
		$\hat{a}$	0.11292^{1}^	0.12916^{6}^	0.12887^{5}^	0.11948^{3}^	0.12301^{4}^	0.13899^{8}^	0.13016^{7}^	0.11928^{2}^
	MSE	$\hat{\nu }$	0.00836^{7}^	0.0075^{3}^	0.00741^{2}^	0.00864^{8}^	0.00691^{1}^	0.00823^{6}^	0.00756^{4}^	0.00768^{5}^
		$\hat{a}$	0.0199^{1}^	0.02582^{5}^	0.02802^{7}^	0.02191^{2}^	0.0241^{4}^	0.02878^{8}^	0.02617^{6}^	0.02245^{3}^
	MRE	$\hat{\nu }$	0.29407^{7}^	0.26245^{3}^	0.25731^{2}^	0.29606^{8}^	0.24636^{1}^	0.28505^{6}^	0.26873^{5}^	0.26777^{4}^
		$\hat{a}$	0.07528^{1}^	0.08611^{6}^	0.08591^{5}^	0.07965^{3}^	0.08201^{4}^	0.09266^{8}^	0.08677^{7}^	0.07952^{2}^
	∑ *Ranks*		24^{4}^	26^{5}^	23^{3}^	32^{6}^	15^{1}^	42^{8}^	34^{7}^	20^{2}^
125	BIAS	$\hat{\nu }$	0.06375^{7}^	0.05988^{4}^	0.05211^{1}^	0.06511^{8}^	0.0572^{2}^	0.06119^{6}^	0.05961^{3}^	0.06084^{5}^
		$\hat{a}$	0.10119^{1}^	0.11089^{6}^	0.10888^{4}^	0.10445^{2}^	0.11059^{5}^	0.12703^{8}^	0.12391^{7}^	0.10883^{3}^
	MSE	$\hat{\nu }$	0.00655^{7}^	0.00604^{4}^	0.00488^{1}^	0.00665^{8}^	0.00562^{2}^	0.00636^{5}^	0.00579^{3}^	0.00639^{6}^
		$\hat{a}$	0.01605^{1}^	0.01937^{4}^	0.02008^{6}^	0.01664^{2}^	0.01988^{5}^	0.02363^{7}^	0.0247^{8}^	0.01845^{3}^
	MRE	$\hat{\nu }$	0.25498^{7}^	0.23952^{4}^	0.20842^{1}^	0.26045^{8}^	0.22881^{2}^	0.24475^{6}^	0.23844^{3}^	0.24337^{5}^
		$\hat{a}$	0.06746^{1}^	0.07393^{6}^	0.07259^{4}^	0.06963^{2}^	0.07372^{5}^	0.08469^{8}^	0.08261^{7}^	0.07255^{3}^
	∑ *Ranks*		24^{3}^	28^{5}^	17^{1}^	30^{6}^	21^{2}^	40^{8}^	31^{7}^	25^{4}^
150	BIAS	$\hat{\nu }$	0.05766^{6}^	0.05125^{2}^	0.04978^{1}^	0.06057^{8}^	0.05205^{3}^	0.06007^{7}^	0.05325^{4}^	0.05569^{5}^
		$\hat{a}$	0.08878^{1}^	0.1036^{5}^	0.10562^{7}^	0.0946^{2}^	0.10345^{4}^	0.11287^{8}^	0.10479^{6}^	0.10163^{3}^
	MSE	$\hat{\nu }$	0.00538^{6}^	0.00466^{2.5}^	0.00453^{1}^	0.0057^{7}^	0.00477^{4}^	0.00596^{8}^	0.00466^{2.5}^	0.00537^{5}^
		$\hat{a}$	0.01258^{1}^	0.01697^{4}^	0.01816^{7}^	0.01393^{2}^	0.01722^{5}^	0.0191^{8}^	0.0174^{6}^	0.01618^{3}^
	MRE	$\hat{\nu }$	0.23064^{6}^	0.205^{2}^	0.19912^{1}^	0.24227^{8}^	0.2082^{3}^	0.2403^{7}^	0.21302^{4}^	0.22276^{5}^
		$\hat{a}$	0.05919^{1}^	0.06906^{5}^	0.07041^{7}^	0.06307^{2}^	0.06897^{4}^	0.07525^{8}^	0.06986^{6}^	0.06775^{3}^
	∑ *Ranks*		21^{2}^	20.5^{1}^	24^{4.5}^	29^{7}^	23^{3}^	46^{8}^	28.5^{6}^	24^{4.5}^

**Table 4:** Simulation values of BIAS, MSE; MRE for (*ν* = 0.5, *a* = −2.5).

n	Est.	Est. Par.	*EM*1	*EM*2	*EM*3	*EM*4	*EM*5	*EM*6	*EM*7	*EM*8
25	BIAS	$\hat{\nu }$	0.07941^{8}^	0.07544^{5}^	0.07068^{3}^	0.0695^{2}^	0.06852^{1}^	0.07593^{7}^	0.07107^{4}^	0.07583^{6}^
		$\hat{a}$	0.41494^{3}^	0.40053^{1}^	0.47532^{7}^	0.42768^{5}^	0.4373^{6}^	0.40831^{2}^	0.48613^{8}^	0.42178^{4}^
	MSE	$\hat{\nu }$	0.01022^{8}^	0.00933^{6}^	0.00904^{4}^	0.00767^{1}^	0.00797^{2}^	0.00962^{7}^	0.00862^{3}^	0.00918^{5}^
		$\hat{a}$	0.28955^{4}^	0.26986^{2}^	0.42849^{8}^	0.26998^{3}^	0.33426^{6}^	0.26014^{1}^	0.42202^{7}^	0.31277^{5}^
	MRE	$\hat{\nu }$	0.15881^{8}^	0.15087^{5}^	0.14135^{3}^	0.13901^{2}^	0.13704^{1}^	0.15185^{7}^	0.14214^{4}^	0.15167^{6}^
		$\hat{a}$	0.16598^{3}^	0.16021^{1}^	0.19013^{7}^	0.17107^{5}^	0.17492^{6}^	0.16332^{2}^	0.19445^{8}^	0.16871^{4}^
	∑ *Ranks*		34^{7.5}^	20^{2}^	32^{6}^	18^{1}^	22^{3}^	26^{4}^	34^{7.5}^	30^{5}^
50	BIAS	$\hat{\nu }$	0.05549^{7}^	0.05593^{8}^	0.04692^{1}^	0.05297^{3}^	0.04899^{2}^	0.05319^{4}^	0.05374^{5}^	0.05536^{6}^
		$\hat{a}$	0.26971^{1}^	0.29246^{4}^	0.31307^{7}^	0.2848^{2}^	0.29922^{6}^	0.2983^{5}^	0.3154^{8}^	0.28823^{3}^
	MSE	$\hat{\nu }$	0.00485^{5.5}^	0.00496^{8}^	0.00388^{1}^	0.00438^{3}^	0.00418^{2}^	0.00485^{5.5}^	0.00491^{7}^	0.00484^{4}^
		$\hat{a}$	0.11876^{1}^	0.13948^{4}^	0.1683^{7}^	0.12455^{2}^	0.14884^{6}^	0.13451^{3}^	0.17764^{8}^	0.14137^{5}^
	MRE	$\hat{\nu }$	0.11099^{7}^	0.11186^{8}^	0.09383^{1}^	0.10594^{3}^	0.09799^{2}^	0.10637^{4}^	0.10749^{5}^	0.11071^{6}^
		$\hat{a}$	0.10788^{1}^	0.11698^{4}^	0.12523^{7}^	0.11392^{2}^	0.11969^{6}^	0.11932^{5}^	0.12616^{8}^	0.11529^{3}^
	∑ *Ranks*		22.5^{2}^	36^{7}^	24^{3.5}^	15^{1}^	24^{3.5}^	26.5^{5}^	41^{8}^	27^{6}^
75	BIAS	$\hat{\nu }$	0.04356^{4}^	0.04369^{5}^	0.04036^{1}^	0.04446^{7}^	0.04141^{2}^	0.0446^{8}^	0.04295^{3}^	0.04428^{6}^
		$\hat{a}$	0.23868^{4}^	0.2328^{3}^	0.25919^{7}^	0.22625^{1}^	0.24172^{5}^	0.2458^{6}^	0.2822^{8}^	0.22869^{2}^
	MSE	$\hat{\nu }$	0.00305^{5}^	0.00301^{4}^	0.00271^{1}^	0.00308^{6}^	0.00291^{2}^	0.00338^{8}^	0.00296^{3}^	0.00317^{7}^
		$\hat{a}$	0.09148^{4}^	0.08877^{3}^	0.11127^{7}^	0.07738^{1}^	0.09526^{6}^	0.09246^{5}^	0.13762^{8}^	0.08503^{2}^
	MRE	$\hat{\nu }$	0.08713^{4}^	0.08739^{5}^	0.08072^{1}^	0.08892^{7}^	0.08281^{2}^	0.08921^{8}^	0.08591^{3}^	0.08855^{6}^
		$\hat{a}$	0.09547^{4}^	0.09312^{3}^	0.10368^{7}^	0.0905^{1}^	0.09669^{5}^	0.09832^{6}^	0.11288^{8}^	0.09148^{2}^
	∑ *Ranks*		25^{5.5}^	23^{2.5}^	24^{4}^	23^{2.5}^	22^{1}^	41^{8}^	33^{7}^	25^{5.5}^
100	BIAS	$\hat{\nu }$	0.04008^{8}^	0.0392^{6}^	0.03657^{2}^	0.03824^{4}^	0.03708^{3}^	0.03864^{5}^	0.03568^{1}^	0.0396^{7}^
		$\hat{a}$	0.19302^{1}^	0.20514^{4}^	0.2119^{5}^	0.20448^{3}^	0.21653^{7}^	0.21368^{6}^	0.22662^{8}^	0.19953^{2}^
	MSE	$\hat{\nu }$	0.00248^{6}^	0.00242^{5}^	0.00224^{2}^	0.00228^{3.5}^	0.00228^{3.5}^	0.00254^{7}^	0.00213^{1}^	0.00257^{8}^
		$\hat{a}$	0.059^{1}^	0.06835^{4}^	0.07688^{7}^	0.06486^{3}^	0.07455^{6}^	0.06905^{5}^	0.08366^{8}^	0.06318^{2}^
	MRE	$\hat{\nu }$	0.08016^{8}^	0.07839^{6}^	0.07313^{2}^	0.07649^{4}^	0.07416^{3}^	0.07729^{5}^	0.07137^{1}^	0.07921^{7}^
		$\hat{a}$	0.07721^{1}^	0.08205^{4}^	0.08476^{5}^	0.08179^{3}^	0.08661^{7}^	0.08547^{6}^	0.09065^{8}^	0.07981^{2}^
	∑ *Ranks*		25^{3}^	29^{6}^	23^{2}^	20.5^{1}^	29.5^{7}^	34^{8}^	27^{4}^	28^{5}^
125	BIAS	$\hat{\nu }$	0.03551^{7}^	0.03258^{2}^	0.03308^{3}^	0.03456^{5}^	0.03164^{1}^	0.035^{6}^	0.03566^{8}^	0.03453^{4}^
		$\hat{a}$	0.16567^{1}^	0.17793^{2}^	0.19364^{5}^	0.1805^{3}^	0.19523^{6}^	0.20227^{8}^	0.19784^{7}^	0.19063^{4}^
	MSE	$\hat{\nu }$	0.00196^{6}^	0.00173^{1.5}^	0.00181^{3}^	0.00182^{4}^	0.00173^{1.5}^	0.00198^{7}^	0.00203^{8}^	0.00188^{5}^
		$\hat{a}$	0.0451^{1}^	0.05185^{3}^	0.06246^{7}^	0.0499^{2}^	0.06185^{5}^	0.06243^{6}^	0.06458^{8}^	0.05787^{4}^
	MRE	$\hat{\nu }$	0.07101^{7}^	0.06515^{2}^	0.06617^{3}^	0.06911^{5}^	0.06328^{1}^	0.07001^{6}^	0.07132^{8}^	0.06907^{4}^
		$\hat{a}$	0.06627^{1}^	0.07117^{2}^	0.07745^{5}^	0.0722^{3}^	0.07809^{6}^	0.08091^{8}^	0.07914^{7}^	0.07625^{4}^
	∑ *Ranks*		23^{4}^	12.5^{1}^	26^{6}^	22^{3}^	20.5^{2}^	41^{7}^	46^{8}^	25^{5}^
150	BIAS	$\hat{\nu }$	0.03125^{4}^	0.03256^{7}^	0.02988^{2}^	0.03299^{8}^	0.02927^{1}^	0.03164^{6}^	0.03033^{3}^	0.03156^{5}^
		$\hat{a}$	0.16156^{2}^	0.16064^{1}^	0.17681^{7}^	0.16669^{5}^	0.16668^{4}^	0.17385^{6}^	0.18209^{8}^	0.16167^{3}^
	MSE	$\hat{\nu }$	0.00155^{4}^	0.00162^{5.5}^	0.00151^{2}^	0.00171^{8}^	0.00147^{1}^	0.00165^{7}^	0.00153^{3}^	0.00162^{5.5}^
		$\hat{a}$	0.0404^{1}^	0.04202^{2}^	0.05017^{7}^	0.043^{3}^	0.04525^{5}^	0.04589^{6}^	0.05594^{8}^	0.04312^{4}^
	MRE	$\hat{\nu }$	0.0625^{4}^	0.06512^{7}^	0.05976^{2}^	0.06599^{8}^	0.05854^{1}^	0.06328^{6}^	0.06067^{3}^	0.06312^{5}^
		$\hat{a}$	0.06463^{2}^	0.06426^{1}^	0.07072^{7}^	0.06668^{5}^	0.06667^{4}^	0.06954^{6}^	0.07283^{8}^	0.06467^{3}^
	∑ *Ranks*		17^{2}^	23.5^{3}^	27^{5}^	37^{7.5}^	16^{1}^	37^{7.5}^	33^{6}^	25.5^{4}^

**Table 5:** Simulation values of BIAS, MSE; MRE for (*ν* = 0.25, *a* = 2.0).

n	Est.	Est. Par.	*EM*1	*EM*2	*EM*3	*EM*4	*EM*5	*EM*6	*EM*7	*EM*8
25	BIAS	$\hat{\nu }$	0.10683^{7}^	0.09906^{4}^	0.09043^{1}^	0.11311^{8}^	0.0939^{2}^	0.10542^{6}^	0.09493^{3}^	0.10039^{5}^
		$\hat{a}$	0.31219^{2}^	0.3071^{1}^	0.38293^{8}^	0.33482^{4}^	0.35588^{6}^	0.32968^{3}^	0.37299^{7}^	0.3443^{5}^
	MSE	$\hat{\nu }$	0.01833^{6}^	0.0169^{4}^	0.01443^{1}^	0.02012^{8}^	0.01536^{2}^	0.019^{7}^	0.01601^{3}^	0.01704^{5}^
		$\hat{a}$	0.17023^{4}^	0.15962^{1}^	0.26771^{8}^	0.16541^{3}^	0.21164^{6}^	0.15978^{2}^	0.24872^{7}^	0.19668^{5}^
	MRE	$\hat{\nu }$	0.42733^{7}^	0.39623^{4}^	0.36171^{1}^	0.45244^{8}^	0.37558^{2}^	0.42167^{6}^	0.37972^{3}^	0.40155^{5}^
		$\hat{a}$	0.15609^{2}^	0.15355^{1}^	0.19147^{8}^	0.16741^{4}^	0.17794^{6}^	0.16484^{3}^	0.1865^{7}^	0.17215^{5}^
	∑ *Ranks*		28^{5}^	15^{1}^	27^{3.5}^	35^{8}^	24^{2}^	27^{3.5}^	30^{6.5}^	30^{6.5}^
50	BIAS	$\hat{\nu }$	0.07626^{8}^	0.07003^{3}^	0.0636^{2}^	0.076^{7}^	0.06169^{1}^	0.07188^{5}^	0.07054^{4}^	0.07506^{6}^
		$\hat{a}$	0.22491^{3}^	0.22385^{2}^	0.23793^{6}^	0.21728^{1}^	0.22928^{4}^	0.24747^{7}^	0.26378^{8}^	0.23707^{5}^
	MSE	$\hat{\nu }$	0.00928^{7}^	0.00835^{4}^	0.00718^{2}^	0.00906^{6}^	0.007^{1}^	0.00901^{5}^	0.00833^{3}^	0.00933^{8}^
		$\hat{a}$	0.08174^{2}^	0.08581^{3}^	0.0973^{7}^	0.07054^{1}^	0.08674^{4}^	0.09019^{5}^	0.11454^{8}^	0.09048^{6}^
	MRE	$\hat{\nu }$	0.30504^{8}^	0.28011^{3}^	0.25439^{2}^	0.30399^{7}^	0.24677^{1}^	0.28754^{5}^	0.28215^{4}^	0.30025^{6}^
		$\hat{a}$	0.11245^{3}^	0.11193^{2}^	0.11896^{6}^	0.10864^{1}^	0.11464^{4}^	0.12374^{7}^	0.13189^{8}^	0.11854^{5}^
	∑ *Ranks*		31^{5}^	17^{2}^	25^{4}^	23^{3}^	15^{1}^	34^{6}^	35^{7}^	36^{8}^
75	BIAS	$\hat{\nu }$	0.06264^{7}^	0.05901^{4}^	0.05267^{1}^	0.06637^{8}^	0.0534^{2}^	0.0594^{6}^	0.05643^{3}^	0.05932^{5}^
		$\hat{a}$	0.17586^{2}^	0.18908^{3}^	0.19645^{6}^	0.17511^{1}^	0.19392^{5}^	0.20788^{7}^	0.21263^{8}^	0.19124^{4}^
	MSE	$\hat{\nu }$	0.00634^{7}^	0.00596^{5}^	0.00506^{1}^	0.0068^{8}^	0.00516^{2}^	0.00599^{6}^	0.00541^{3}^	0.00586^{4}^
		$\hat{a}$	0.05053^{2}^	0.05683^{4}^	0.06539^{7}^	0.04655^{1}^	0.06013^{5}^	0.06364^{6}^	0.07264^{8}^	0.05587^{3}^
	MRE	$\hat{\nu }$	0.25055^{7}^	0.23604^{4}^	0.21069^{1}^	0.26546^{8}^	0.21359^{2}^	0.23762^{6}^	0.22571^{3}^	0.23729^{5}^
		$\hat{a}$	0.08793^{2}^	0.09454^{3}^	0.09823^{6}^	0.08755^{1}^	0.09696^{5}^	0.10394^{7}^	0.10632^{8}^	0.09562^{4}^
	∑ *Ranks*		27^{5.5}^	23^{3}^	22^{2}^	27^{5.5}^	21^{1}^	38^{8}^	33^{7}^	25^{4}^
100	BIAS	$\hat{\nu }$	0.05469^{7}^	0.05202^{5}^	0.04715^{2}^	0.05705^{8}^	0.04626^{1}^	0.05286^{6}^	0.0503^{4}^	0.0501^{3}^
		$\hat{a}$	0.15042^{1}^	0.15299^{2}^	0.1681^{6}^	0.15437^{3}^	0.16043^{5}^	0.18109^{8}^	0.17518^{7}^	0.15775^{4}^
	MSE	$\hat{\nu }$	0.00465^{6}^	0.00458^{5}^	0.00391^{2}^	0.00516^{8}^	0.0039^{1}^	0.0047^{7}^	0.00422^{4}^	0.00416^{3}^
		$\hat{a}$	0.03671^{1}^	0.03803^{3}^	0.04662^{6}^	0.03697^{2}^	0.04148^{5}^	0.04694^{7}^	0.05087^{8}^	0.03968^{4}^
	MRE	$\hat{\nu }$	0.21876^{7}^	0.20807^{5}^	0.18859^{2}^	0.22819^{8}^	0.18502^{1}^	0.21143^{6}^	0.20119^{4}^	0.20042^{3}^
		$\hat{a}$	0.07521^{1}^	0.07649^{2}^	0.08405^{6}^	0.07719^{3}^	0.08021^{5}^	0.09055^{8}^	0.08759^{7}^	0.07888^{4}^
	∑ *Ranks*		23^{4}^	22^{3}^	24^{5}^	32^{6}^	18^{1}^	42^{8}^	34^{7}^	21^{2}^
125	BIAS	$\hat{\nu }$	0.05021^{7}^	0.04541^{4}^	0.04256^{1}^	0.05093^{8}^	0.04407^{2}^	0.04738^{6}^	0.04567^{5}^	0.04447^{3}^
		$\hat{a}$	0.13241^{1}^	0.1406^{3}^	0.1464^{5}^	0.1358^{2}^	0.15082^{7}^	0.15874^{8}^	0.14905^{6}^	0.14234^{4}^
	MSE	$\hat{\nu }$	0.00386^{6}^	0.00341^{4}^	0.0032^{1}^	0.00394^{8}^	0.00339^{3}^	0.00388^{7}^	0.00356^{5}^	0.00333^{2}^
		$\hat{a}$	0.02827^{2}^	0.03219^{4}^	0.03485^{6}^	0.02713^{1}^	0.03477^{5}^	0.03798^{8}^	0.03713^{7}^	0.0319^{3}^
	MRE	$\hat{\nu }$	0.20082^{7}^	0.18164^{4}^	0.17023^{1}^	0.20372^{8}^	0.17627^{2}^	0.18951^{6}^	0.1827^{5}^	0.17789^{3}^
		$\hat{a}$	0.06621^{1}^	0.0703^{3}^	0.0732^{5}^	0.0679^{2}^	0.07541^{7}^	0.07937^{8}^	0.07452^{6}^	0.07117^{4}^
	∑ *Ranks*		24^{4}^	22^{3}^	19^{1.5}^	29^{6}^	26^{5}^	43^{8}^	34^{7}^	19^{1.5}^
150	BIAS	$\hat{\nu }$	0.04628^{8}^	0.04182^{3}^	0.04013^{2}^	0.04382^{7}^	0.03963^{1}^	0.04246^{5}^	0.04222^{4}^	0.04352^{6}^
		$\hat{a}$	0.12304^{2}^	0.13286^{5}^	0.13275^{4}^	0.12199^{1}^	0.13266^{3}^	0.15185^{8}^	0.14755^{7}^	0.13595^{6}^
	MSE	$\hat{\nu }$	0.00333^{8}^	0.00287^{2}^	0.00298^{4}^	0.00305^{5}^	0.00266^{1}^	0.0031^{7}^	0.00296^{3}^	0.00309^{6}^
		$\hat{a}$	0.02452^{2}^	0.02809^{4}^	0.02877^{6}^	0.02339^{1}^	0.02734^{3}^	0.03371^{7}^	0.03435^{8}^	0.02859^{5}^
	MRE	$\hat{\nu }$	0.1851^{8}^	0.1673^{3}^	0.16051^{2}^	0.17526^{7}^	0.15854^{1}^	0.16983^{5}^	0.16889^{4}^	0.17407^{6}^
		$\hat{a}$	0.06152^{2}^	0.06643^{5}^	0.06637^{4}^	0.06099^{1}^	0.06633^{3}^	0.07592^{8}^	0.07378^{7}^	0.06797^{6}^
	∑ *Ranks*		30^{5}^	22^{3}^	22^{3}^	22^{3}^	12^{1}^	40^{8}^	33^{6}^	35^{7}^

**Table 6:** Partial and overall ranks of all the methods of estimation of proposed distribution by various values of model parameters.

Parameter	*n*	*EM*1	*EM*2	*EM*3	*EM*4	*EM*5	*EM*6	*EM*7	*EM*8
*ν* = −0.5, *a* = −0.75	25	8.0	4.0	2.0	5.0	1.0	7.0	6.0	3.0
	50	8.0	4.0	2.0	6.0	1.0	7.0	5.0	3.0
	75	7.0	6.0	1.5	5.0	1.5	8.0	4.0	3.0
	100	6.0	3.0	2.0	7.0	1.0	8.0	4.0	5.0
	125	7.0	3.0	2.0	6.0	1.0	8.0	5.0	4.0
	150	6.0	4.0	1.0	8.0	2.0	7.0	5.0	3.0
*ν* = 0.75, *a* = 0.5	25	4.0	6.0	5.0	1.0	2.0	3.0	7.5	7.5
	50	5.0	6.0	3.0	2.0	1.0	8.0	7.0	4.0
	75	6.0	5.0	1.0	2.0	3.0	7.0	8.0	4.0
	100	5.5	4.0	3.0	1.5	1.5	7.0	8.0	5.5
	125	4.0	5.0	2.0	3.0	1.0	8.0	7.0	6.0
	150	5.5	4.0	1.0	3.0	2.0	8.0	5.5	7.0
*ν* = −0.25, *a* = 1.5	25	2.0	5.0	3.0	6.0	1.0	8.0	7.0	4.0
	50	3.5	2.0	3.5	6.0	1.0	7.0	8.0	5.0
	75	6.0	3.5	5.0	3.5	1.0	8.0	7.0	2.0
	100	4.0	5.0	3.0	6.0	1.0	8.0	7.0	2.0
	125	3.0	5.0	1.0	6.0	2.0	8.0	7.0	4.0
	150	2.0	1.0	4.5	7.0	3.0	8.0	6.0	4.5
*ν* = 0.5, *a* = −2.5	25	7.5	2.0	6.0	1.0	3.0	4.0	7.5	5.0
	50	2.0	7.0	3.5	1.0	3.5	5.0	8.0	6.0
	75	5.5	2.5	4.0	2.5	1.0	8.0	7.0	5.5
	100	3.0	6.0	2.0	1.0	7.0	8.0	4.0	5.0
	125	4.0	1.0	6.0	3.0	2.0	7.0	8.0	5.0
	150	2.0	3.0	5.0	7.5	1.0	7.5	6.0	4.0
*ν* = 0.25, *a* = 2.0	25	5.0	1.0	3.5	8.0	2.0	3.5	6.5	6.5
	50	5.0	2.0	4.0	3.0	1.0	6.0	7.0	8.0
	75	5.5	3.0	2.0	5.5	1.0	8.0	7.0	4.0
	100	4.0	3.0	5.0	6.0	1.0	8.0	7.0	2.0
	125	4.0	3.0	1.5	6.0	5.0	8.0	7.0	1.5
	150	5.0	3.0	3.0	3.0	1.0	8.0	6.0	7.0
∑ Ranks		145.0	112.0	91.0	131.5	55.5	214.0	195.0	136.0
Overall Rank		6	3	2	4	1	8	7	5

From the simulation study and ranking tables, we conclude that:

- All of the estimators show the consistency characteristic.
- For all estimation methods, the BIAS of all estimators reduces as *n* increases.
- For all estimation methods, the MSE of all estimators reduces as *n* increases.
- For all estimation methods, the MRE of all estimators reduces as *n* increases.
- The least-squares estimation method is the best method for estimating the considered parameters. So, we advise researchers to use this method if they have data sets from our proposed model.

## 6 Real data analysis

Here, we consider the Bitcoin real data set to depict the flexibility of the proposed distribution. It represents the daily Bitcoin ERs based on the USA dollars from June 30, 2014, to June 30, 2022. It is available on (https://coinmarketcap.com/currencies/bitcoin/), and it also studied by Zubair et al. [23].

To show how adaptable the considered models are, we will analyze the suggested model in contrast to a variety of well-known models, such as type IV generalized logistic distribution (TIVGLD) [24], logistic distribution (LD), Gumbel distribution (GD), Arctan Gumbel distribution (AGD) [25], normal distribution (ND), Arctan Normal distribution (AND) [25], beta generalized logistic type IV distribution (BGLTIVD) [26], and type II beta generalized logistic distribution (TIIBGLD) [27].

The computed models were compared utilizing some analytical measures, including some information criteria (IC), like Akaike IC (AIC), corrected AIC (CAIC), and Hannan–Quinn IC (HQIC), through some goodness-of-fit measures, such as Cramer–von Mises (CM), Anderson–Darling (AD) and Kolmogorov–Smirnov (KS) with its *p*-value (KS *p*-value) to determine the best-fitting model for the considered real data sets. The analytical measures, MLE, and associated standard errors (SEs) are provided in parentheses for the Bitcoin real data set in Table 7. From this table, we can conclude that the HS model fits better than other models in that it has lower values for all measures except for KS *p*-value.

**Table 7:** Numerical information criteria values with the goodness-of-fit measures for the Bitcoin real data set.

Model	AIC	CAIC	BIC	HQIC	AD	CM	KS	KS *p*-value	Est. parameters (SEs)
HSD	-1345.43	-1345.4	-1337.73	-1342.37	0.301266	0.0412091	0.0298074	0.916611	$\hat{\nu }=-0.000269335\;\left(0.00171984\right)$
									$\hat{a}=43.8134\;\left(2.11985\right)$
TIVGLD	-1160.73	-1160.7	-1153.03	-1157.66	29.9558	5.53338	0.223352	<0.00001	$\hat{p}=520.631\;\left(39.4595\right)$
									$\hat{q}=515.665\;\left(39.0829\right)$
LD	-1341.95	-1341.91	-1334.24	-1338.88	0.493491	0.0771213	0.0373416	0.716889	$\hat{\eta }=-0.000554922\;\left(0.00178228\right)$
									$\hat{\lambda }=0.0193543\;\left(0.000875427\right)$
GD	-1172.8	-1172.77	-1165.1	-1169.74	15.7443	2.60965	0.132164	0.0000104999	$\hat{\eta }=-0.0202079\;\left(0.00150616\right)$
									$\hat{\lambda }=0.0444967\;\left(0.0041604\right)$
AGD	-1187.59	-1187.56	-1179.89	-1184.53	13.9623	2.24874	0.132372	0.0000101057	$\hat{\eta }=-0.00896221\;\left(0.00254034\right)$
									$\hat{\lambda }=0.0485941\;\left(0.00166814\right)$
ND	-1317.36	-1317.33	-1309.66	-1314.29	2.09909	0.366212	0.0625219	0.131625	$\hat{\eta }=-0.00145123\;\left(0.00305665\right)$
									$\hat{\lambda }=0.0362453\;\left(0.0156367\right)$
AND	-1318.87	-1318.84	-1311.17	-1315.81	1.96055	0.328475	0.0618034	0.14006	$\hat{\eta }=0.00647634\;\left(0.001968\right)$
									$\hat{\lambda }=0.0374487\;\left(0.00145664\right)$
BGLTIVD	-1344.35	-1344.28	-1332.79	-1339.75	0.308089	0.0582173	0.0380846	0.69376	$\hat{\mu }=117.885\;\left(65.4528\right)$
									$\hat{\sigma }=0.342706\;\left(0.21917\right)$
									$\hat{a}=0.361406\;\left(0.230347\right)$
TIIBGLD	-1170.98	-1170.91	-1159.42	-1166.38	28.2496	5.20011	0.217225	<0.00001	$\hat{q}=1.27689\;\left(0.0131009\right)$
									$\hat{a}=562.656\;\left(24.5382\right)$
									$\hat{p}=391.546\;\left(9.28509\right)$

**Fig 7:**
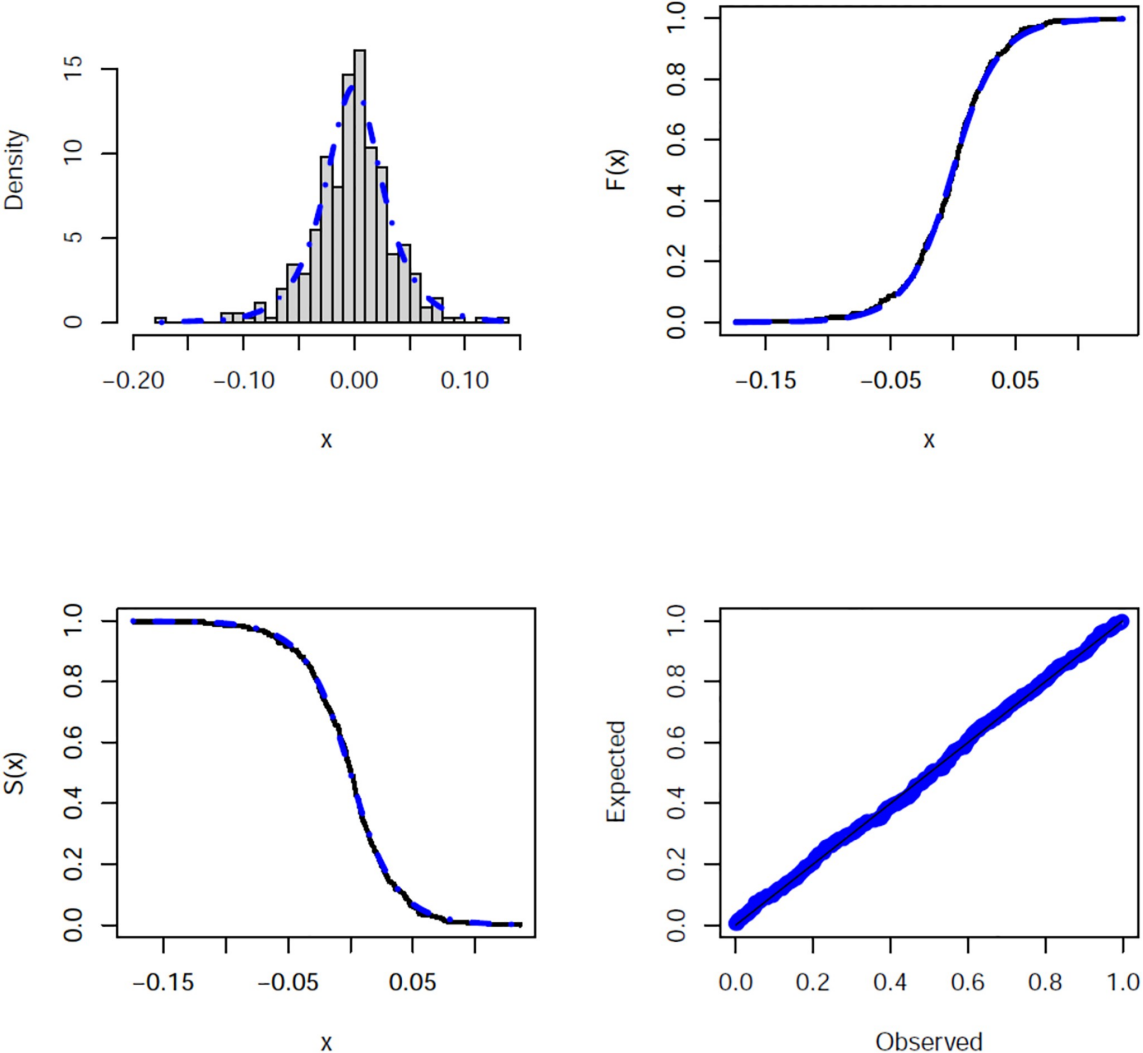
Histogram of the Bitcoin real data set with the fitted PDF, CDF, SF, and P-P plots.

**Fig 8:**
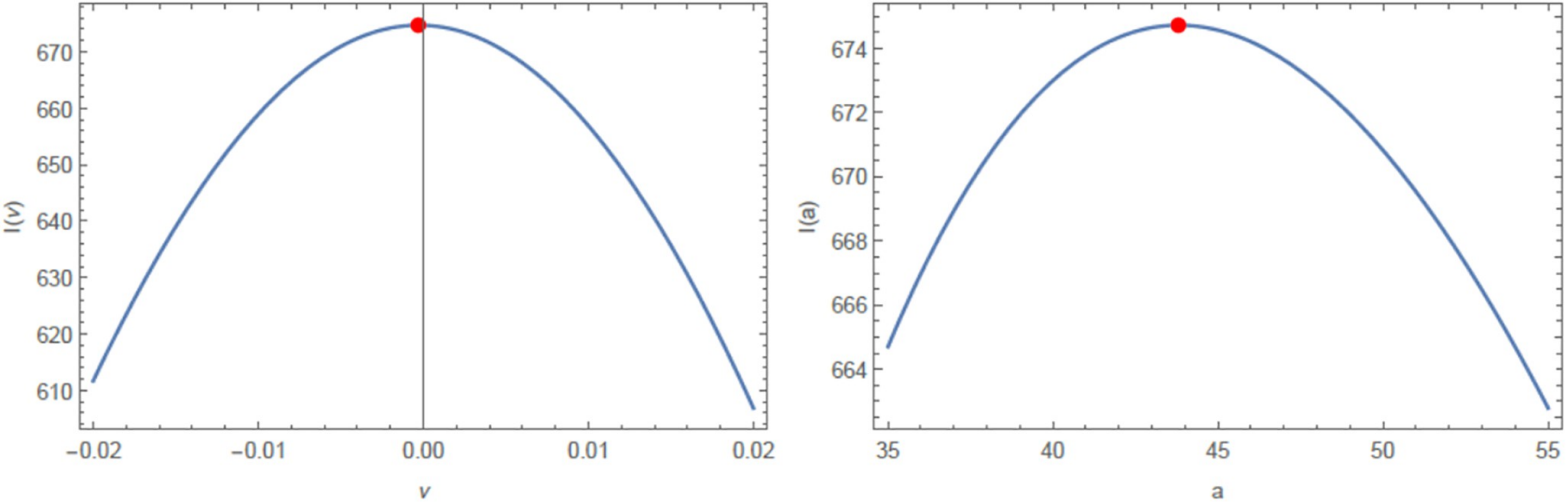
The profile of the log-likelihood functions for *ν* and *a* of the Bitcoin real data set.

Fig 7 illustrates the estimated PDF, CDF, SF, and P-P plots of the HS distribution for the Bitcoin real data set. These figures confirm that HS distribution is superior for fitting the real data set. Fig 8 provides the behavior of the log-likelihood function with estimated parameters, which present an unimodal function and reflect that the estimated parameters are global maximum points. This means that they maximize the log-likelihood function, giving the best estimate of the parameters.

## 7 Conclusions

In the present study, we generated a novel probability density function of the hyperbolic secant (HS) distribution using the nonlinear Phi-4 equation. Namely, we produce this probability density function for the sech solution. In actuality, this density function involves a range of shapes, such as left-skewed, symmetric, and right-skewed. Using eight different frequent estimators for the parameters of our models, the behavior of the HS model parameters was examined using estimation techniques on randomly generated data sets. We also extend our results to real-world data to demonstrate the HS distribution’s utility in simulating real-world data. It is demonstrated that the suggested model is more flexible and offers a better fit to the data when compared to other distributions based on all statistics used in the real data analysis section.

## References

[1] Mirzaee F., Rezaei S. and Samadyar N., Numerical solution of two-dimensional stochastic time-fractional sine-Gordon equation on non-rectangular domains using finite difference and meshfree methods, Engineering Analysis with Boundary Elements, 127 (2021), 53–63. doi: 10.1016/j.enganabound.2021.03.009

[2] Abdelrahman M.A.E., Sohaly M.A. and Alharbi Y.F., Fundamental stochastic solutions for the conformable fractional NLSE with spatiotemporal dispersion via exponential distribution, Physica Scripta, 96, 125223 (2021). doi: 10.1088/1402-4896/ac119c

[3] Yamaguchi R., Analysis of electro-optical behavior in liquid crystal cells with asymmetric anchoring strength, Symmetry, 14, 85 (2022). doi: 10.3390/sym14010085

[4] Shakeel M., Iqbal M. A., Din Q., Ayub Q.M. Hassan and K., New exact solutions for coupled nonlinear system of ion sound and Langmuir waves, Indian J Phys, 94(6) (2020), 885–894. doi: 10.1007/s12648-019-01522-7

[5] Alharbi Y.F., Sohaly M.A. and Abdelrahman M.A.E., Fundamental solutions to the stochastic perturbed nonlinear Schrödinger’s equation via gamma distribution, Results in Physics, 25 (2021), 104249. doi: 10.1016/j.rinp.2021.104249

[6] Younas U., Rezazadeh H., Ren J. and Bilal M., Propagation of diverse exact solitary wave solutions in separation phase of iron (Fe-Cr-X(X = Mo, Cu)) for the ternary alloys, International Journal of Modern Physics B, 36(04), 2250039 (2022). doi: 10.1142/S0217979222500394

[7] Stuart A.M., Inverse problems: a Bayesian perspective, Acta Numer., 19 (2010), 451–559. doi: 10.1017/S0962492910000061

[8] Alshreef M.A.E. Abdelrahman and G., Closed-form solutions to the new coupled Konno–Oono equation and the Kaup-Newell model equation in magnetic field with novel statistic application, Eur. Phys. J. Plus 136 (4) (2021), 1–10.

[9] Caraballo T., Ngoc T.B., Thach T.N. and Tuan N.H., On a stochastic nonclassical diffusion equation with standard and fractional Brownian motion, Stochastics and Dynamics, 22(02), 2140011 (2022). doi: 10.1142/S0219493721400116

[10] Ehsani F., Ehsani E., Hadi A. and Hadi N., Analytical solution of phi-four equation, Tech. J. Eng. Applied Sci., 3 (2013), 1378–1388.

[11] Sirisubtawee S., Koonprasert S., Leekparn S. Sungnul and T., Exact traveling wave solutions of the space–time fractional complex Ginzburg–Landau equation and the space-time fractional Phi-4 equation using reliable methods, Adv Differ Equ., 2019, 219 (2019). doi: 10.1186/s13662-019-2154-9

[12] Ivanov R.V., The Semi-Hyperbolic Distribution and Its Applications. Stats, 6(4) (2023), pp.1126–1146. doi: 10.3390/stats6040071

[13] Konlack Socgnia V. and Wilcox D., A comparison of generalized hyperbolic distribution models for equity returns. Journal of Applied Mathematics, 2014(1) (2014), p.263465.

[14] Vaughan D. C., The generalized secant hyperbolic distribution and its properties, Communications in Statistics-Theory and Methods, 31(2)(2002):219–38.

[15] Nadarajah S. and Kotz S., Beta trigonometric distributions. Portuguese Economic Journal, (2006), 5(3), 207–224. doi: 10.1007/s10258-006-0013-6

[16] Abate J., Choudhury G. L., Lucantoni D. N. and Whitt W., Asymptotic analysis of tail probabilities based on the computation of moments. The Annals of Applied Probability, (1995), 983–1007.

[17] Abd El-Bar A.M.T., Bakouch H.S. and Chowdhury S., A new trigonometric distribution with bounded support and an application. Revista de la Unión Matemática Argentina, (2021), 62(2), 459–473.

[18] Daghistani A. F., Abd El-Bar A. M., Gemeay A. M., Abdelrahman M. A., Hassan S. Z., A Hyperbolic Secant-Squared Distribution via the Nonlinear Evolution Equation and Its Application, Mathematics, 11(20)(2023):4270. doi: 10.3390/math11204270

[19] Thongchan P., Bodhisuwan W., A modified hyperbolic secant distribution. Songklanakarin J. Sci. Technol, (2017), 39 (1), 11–18.

[20] Alomair R.A., Hassan S.Z. and Abdelrahman M.A.E., A new structure of solutions to the coupled nonlinear Maccari’s systems in plasma physics, AIMS Math., 7(5) (2022), 8588–8606. doi: 10.3934/math.2022479

[21] Al-Babtain A.A., Gemeay A.M., Afify A.Z., Estimation methods for the discrete Poisson-Lindley and discrete Lindley distributions with actuarial measures and applications in medicine. Journal of King Saud University-Science, 33(2), 101224 (2021). doi: 10.1016/j.jksus.2020.10.021

[22] Teamah A.E.A., Elbanna A.A. and Gemeay A.M., Heavy-Tailed Log-Logistic Distribution: Properties, Risk Measures and Applications. Statistics, Optimization & Information Computing, 9(4) (2021), 910–941. doi: 10.19139/soic-2310-5070-1220

[23] Ahmad Z., Almaspoor Z., Khan F., Alhazmi S.E., El-Morshedy M., Ababneh O.Y. et al., On fitting and forecasting the log-returns of cryptocurrency exchange rates using a new logistic model and machine learning algorithms, AIMS Mathematics, 7(10) (2022), 18031–18049. doi: 10.3934/math.2022993

[24] Prentice R.L., A generalization of the probit and logit methods for dose response curves, Biometrics, 32(4) (1976), 761–768. doi: 10.2307/2529262 1009225

[25] Alkhairy I., Nagy M., Muse A.H. and Hussam E., The Arctan-X family of distributions: Properties, simulation, and applications to actuarial sciences. Complexity (2021), 2021. doi: 10.1155/2021/4689010

[26] Nassar M.M. and Elmasry A., A study of generalized logistic distributions. Journal of the Egyptian Mathematical Society, 20(2) (2012), 126–133. doi: 10.1016/j.joems.2012.08.011

[27] Morais A.L. and Cordeiro G.M. and Cysneiros A.H., The beta generalized logistic distribution, Braz. J. Probab. Stat., 27 (2013), 185–200. doi: 10.1214/11-BJPS166

